# Coronary MR angiography in children during systole and diastole using a dual cardiac phase scan of the whole heart

**DOI:** 10.1186/1532-429X-11-S1-P68

**Published:** 2009-01-28

**Authors:** Sergio A Uribe Arancibia, Israel Valverde, Philipp Beerbaum, Aaron Bell, Rene Botnar, Reza Razavi, Tobias Schaeffter, Gerald Greil

**Affiliations:** 1 King's College London, London, UK; 2 Hospital Virgen del Rocio, Seville, Spain

**Keywords:** Congenital Heart Disease, Kawasaki Disease, Great Vessel, Bland Altman Plot, Coronary Segment

## Objective

To investigate the feasibility of dual cardiac phase whole heart MRI to obtain Coronary Magnetic Resonance Angiography (CMRA) at end-systole and mid-diastole in children with Congenital Heart Diseases (CHD).

## Background

In ch ildren the visualization of the course of the coronary arteries is highly desirable for surgical planning and for follow up in CHD and Kawasaki disease. CMRA has been successful applied in adults and recently has been demonstrated in young children and infants. In children with CHD a whole heart CMR approach is of great clinical advantage, since the anatomy of the heart and the great vessels can be evaluated in addition to the coronary arteries. Usually, a 3D volume is obtained either at end-systole or mid-diastole. However, in children with high heart-rates and/or with RR variability it is unclear which phase of the cardiac cycle results in better image quality. Therefore, the simultaneous acquisition of the end-systolic and mid-diastolic phase in conjunction with the whole heart approach allows retrospectively selection of the best image phase for coronary artery visualization without any scan time penalty.

## Methods

Eight children (age = 6.38 ± 4.27, height = 116.57 ± 30.77, weight = 23.57 ± 14.25, Heart-rate = 85.02 ± 8.59) with CHD were scanned under general anesthesia on a 1.5 T MR system (Achieva, Philips Healthcare). The cardiac rest period for end-systole and mid-diastole was determined from a 2D SSFP cine scan with high temporal resolution (TR/TE = 3.1/1.6 ms, flip angle 60°, slice thickness 6 mm, 60 to 80 cardiac phases). A previously developed free-breathing navigator gated 3D SSFP dual cardiac phase sequence [1] was then applied in sagittal orientation for imaging of the whole heart including the coronary arteries and great vessels (TR/TE = 3.4/1.7 ms, flip angle 90°, 60–120 slice, isotropic resolution of 1–1.5 mm^3^, temporal resolution of 60 – 75 ms, Sense of 2 in AP direction). Data was obtained during end-systole and mid-diastole and the acquisition window of the 3D scan was adapted accordingly to the shortest rest period. Images were then reformatted along the major axes of the left and right coronary artery for both cardiac phases. Vessel length, diameter and sharpness of the visualized coronary arteries (RCA, LM, LAD, LCx) were measured using the "SoapBubble" software. Image quality was assessed by two independent observers. Statistical analysis and Bland Altman plots were used to compare the different data sets.

## Results

**Figure 1:**
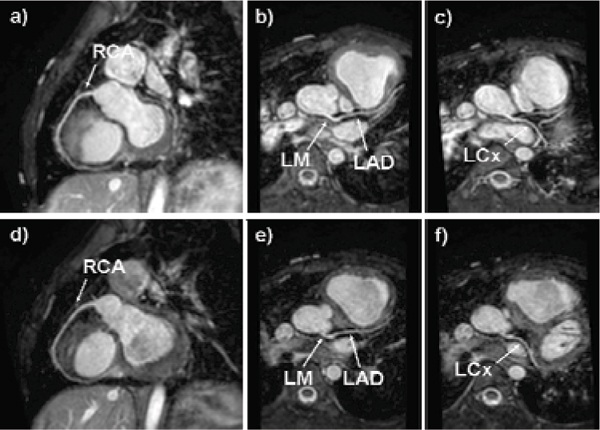
Figure 1

**Figure 2:**
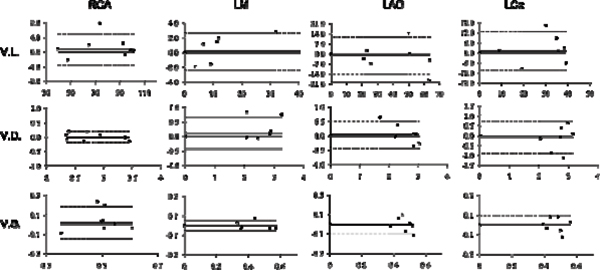
Figure 2

The dual cardiac phase whole heart scan was applied successfully in all patients. An example of each coronary segment is shown in figure 1. The vessel length, diameter, sharpness and consensus score for each segment during systole and diastole are shown in Table 1. Bland Altman plots of the systolic versus diastolic data from each coronary segment are shown in figure 2. No statistically difference was found comparing vessel length, diameter, sharpness for all vessels between systole and diastole. Moreover, no statistically difference was found in image quality. Although, there was no difference for the mean vessel length, diameter, and sharpness, it was found that on a patient level, those parameters and image quality showed differences either favoring systolic or diastolic image acquisition for different coronary segments within the same patient.

**Table 1:** Mean ± SDV values for different parameters

	RCA	RCA	LM	LM	LAD	LAD	LCx	LCx
	Systole	Diastole	Systole	Diastole	Systole	Diastole	Systole	Diastole
Vessel Length	76.94 ± 24.15	75.54 ± 23.96	21.26 ± 31.06	20.93 ± 31.41	41.02 ± 018.44	43.36 ± 20.24	30.35 ± 12.8	28.21 ± 11.48
Vessel Diameter	2.78 ± 0.42	2.76 ± 0.47	2.34 ± 1.15	2.09 ± 1.02	2.58 ± 0.37	2.5 ± 0.64	2.61 ± 0.56	2.77 ± 0.4
Vessel Sharpness	0.52 ± 0.1	0.48 ± 0.09	0.37 ± 0.19	0.37 ± 0.19	0.46 ± 0.05	0.47 ± 0.07	0.47 ± 0.06	0.47 ± 0.07
Consensus Image Quality Score (1 to 4)	3.88 ± 0.44	3.19 ± 0.75	3 ± 0.6	2.5 ± 0.53	3 ± 0.6	2.5 ± 0.53	3.14 ± 0.56	3 ± 1

## Conclusion

A 3D SSFP dual cardiac phase scan of the whole heart was capable of identifying the origin and proximal course of the coronary arteries in children in two cardiac phases. The ability to show the 3D relationship of the heart, great vessels and coronary arteries, and also the possibility to retrospectively select the heart phase with best coronary artery visualization make this technique a clinically valuable tool for surgical planning.
